# Catatonia As Initial Presentation in Anti-N-Methyl-D-Aspartate (NMDA) Receptor Encephalitis: A Case Report of a 19-Year-Old Patient

**DOI:** 10.7759/cureus.74259

**Published:** 2024-11-22

**Authors:** Irving Fuentes-Calvo, Irene Gomez-Oropeza, Adriana Hernandez-Carrasco, Salvador Martinez Medina

**Affiliations:** 1 Neurology, National Institute of Neurology and Neurosurgery "Dr. Manuel Velasco Suárez", Mexico City, MEX

**Keywords:** autoimmune encephalitis, case report, catatonia, neuroinmunology, onset

## Abstract

Anti-N-methyl-D-aspartate receptor (NMDAR) encephalitis stands as the most prevalent form of autoimmune encephalitis, primarily affecting young patients and exhibiting a higher incidence among females. Patients frequently present with psychiatric symptoms or cognitive impairments such as speech disturbances, decreased level of consciousness, autonomic dysfunction, as well as seizures, dyskinesias, and catatonia due to overactivation of extrasynaptic NMDA receptors. To date, there is no gold standard for the diagnosis of catatonia; however, a few rating scales exist to measure this phenomenon, with the Bush Francis Catatonia Rating Scale being the most commonly used due to its validity, reliability, and ease of application. In this case, we present a 19-year-old female who experienced onset symptoms including fluctuating headaches, dysthymia, tinnitus, and prosopagnosia. Eventually, she experienced spatial disorientation and hypoesthesia in the left hemiface, along with insomnia. Subsequently, she reported alterations in perception, accompanied by urinary and fecal incontinence. Upon admission, the patient presented as catatonic. The lumbar puncture revealed normal cell counts, proteins, and glucose levels, along with the presence of reactive anti-NMDAR antibodies in serum and electroencephalogram findings indicating generalized dysfunction, meeting Graus criteria for probable encephalitis. A CT scan revealed a left adnexal mass consistent with an ovarian teratoma. Management commenced with methylprednisolone boluses and plasma exchanges. This case underlines the importance of ongoing research and collaboration in refining treatment strategies for autoimmune encephalitis patients to improve outcomes and quality of life.

## Introduction

Anti-N-methyl-D-aspartate receptor (NMDAR) encephalitis stands as the most prevalent form of autoimmune encephalitis, primarily affecting young patients and exhibiting a higher incidence among females [1]. This immune-mediated disease involves the central nervous system and causes severe inflammation due to multiple antibodies targeting neuronal cell-surface or synaptic proteins, which typically induce selective crosslinking and internalization of these receptors, disrupting the interaction between the NMDAR and the ephrin type B2 (EphB2) receptor. Patients frequently exhibit psychiatric symptoms or cognitive impairment, such as speech disturbances, decreased level of consciousness, autonomic dysfunction, seizures, dyskinesias, and catatonia, due to the overactivation of extrasynaptic NMDARs [2].

Catatonia is a psychomotor disorder that may present with numerous possible symptom combinations, such as pure motor signs (waxy flexibility), disturbances of volition (ambitendence), inability to suppress complex motor activities (stereotypies), and autonomic instability (tachycardia), among others [3]. This frequently occurs in many medical conditions, both psychiatric and non-psychiatric; however, it is more commonly a feature of autoimmune processes, particularly encephalitides associated with antineuronal antibodies, which can cause reversible internalization of the receptor in neurons, inhibiting its function, and making this autoimmune disorder responsible for the majority of cases of catatonia [4]. Common presentations of patients with anti-NMDAR encephalitis without a previous psychiatric medical history include seizures, dyskinesia, disorientation/confusion, and mutism/staring [5]. There is no gold standard for the diagnosis of catatonia; however, a few rating scales exist to measure this phenomenon, with the Bush Francis Catatonia Rating Scale (BFCRS) being the most commonly used to assess the prevalence and quantify its presence due to its validity, reliability, and ease of application. These instruments have been designed to assess the presence or absence of catatonic symptoms and to serve as a screening tool for the syndrome [6].

## Case presentation

The patient is a 19-year-old Mexican female without a significant personal medical history. She reported the onset of symptoms initially characterized by fluctuating headaches, which were managed empirically with nonsteroidal anti-inflammatory drugs (NSAIDs), resulting in partial symptom improvement. Over the subsequent days, she developed a depressed mood, tinnitus, and prosopagnosia. During the evaluation, she described errors in judgment, inattention, and anorexia. One month later, she experienced spatial disorientation along with insomnia. She sought psycho-cognitive therapy due to alterations in perception, predominantly visual hallucinations, accompanied by urinary and fecal incontinence.

**Figure 1:**
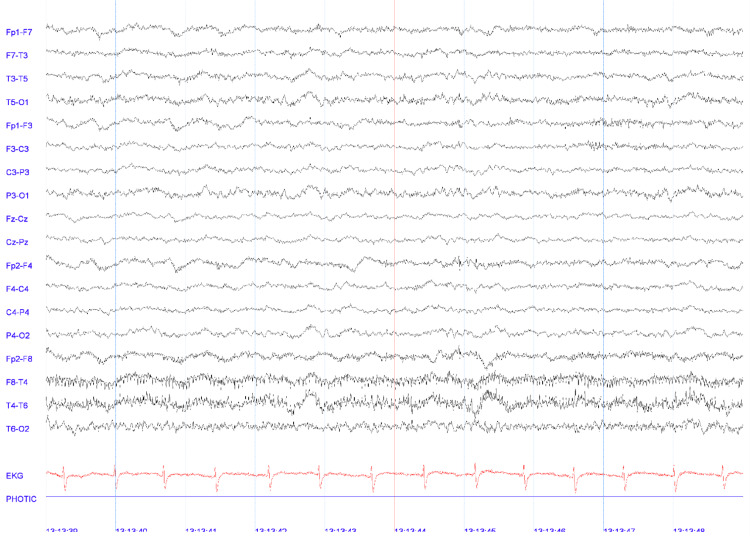
Electroencephalogram. Representative 10-second epoch (double-banana bipolar montage; sensitivity 7 µV/mm; L.F.: 1 Hz, H.F.: 70 Hz) of the patient’s EEG study showing moderate generalized dysfunction without epileptic activity.

One week later, she developed mutism and engaged in soliloquies, leading to admission to our medical unit. Upon admission, the patient presented as catatonic with a Bush and Francis scale score of 4/19, along with hyperreflexia and a bilateral extensor plantar response. General laboratory studies yielded unremarkable findings relevant to the patient's condition. Cranial angiotomography revealed an unruptured left internal carotid artery aneurysm in the clinoid segment. Similarly, a lumbar puncture revealed normal cell counts, proteins, and glucose levels, along with the presence of reactive anti-NMDAR antibodies in the serum. EEG findings indicated generalized dysfunction (Figure 1).

**Figure 2:**
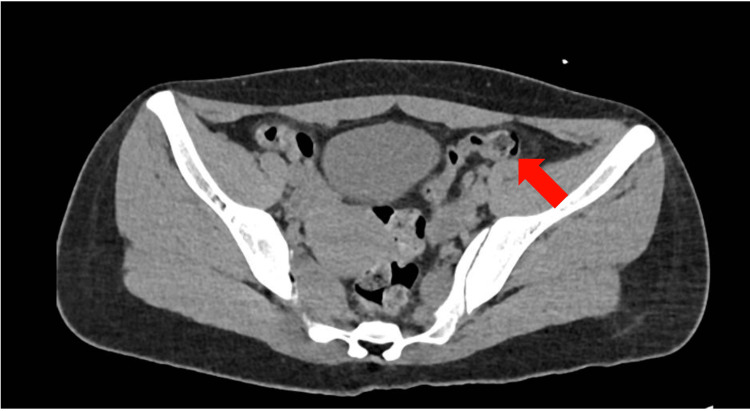
Abdominal CT. Abdominal CT scan revealing enlargement of the left adnexa (volume 11.6 cc) and increased sacrococcygeal angle.

Subsequently, an extended diagnostic approach was pursued with a thoracic and abdominal CT scan, revealing a left adnexal mass consistent with an ovarian teratoma (Figure 2). Management commenced with methylprednisolone boluses (1 gram every 24 hours) followed by plasma exchanges (five sessions). The patient demonstrated clinical improvement, exhibiting interaction with the environment, responsiveness to simple commands, and resolution of hyperreflexia and plantar flexor response. Currently, the patient is undergoing long-term immunomodulatory therapy with Rituximab and has been referred for a neuropsychiatric consultation to address psycho-cognitive needs.

## Discussion

In this case, an unusual presentation is observed, wherein the patient meets the Graus criteria for definite autoimmune encephalitis (AE) as she presented with psychiatric symptoms, accompanied by EEG data showing generalized dysfunction, further confirmed by the presence of serum anti-NMDAR antibodies. This presentation is considered unusual not due to the symptomatology itself, but due to the prematurity of the onset of catatonia in the patient under study. Several studies have noted that the occurrence of catatonia in young patients with AE is rare, and it is even rarer for catatonia to be the initial presentation in these patients [7]. This highlights the first knowledge gap, as most studies have focused on pediatric patients, thereby excluding the remainder of the young population within the second and third decades of life [7,8]. Moreover, catatonia is typically not identified as the initial presentation but rather occurs in more advanced stages of the disease.

A coordinated multidisciplinary approach is essential, addressing clinical manifestations and potentially incorporating psycho-cognitive therapy, alongside the necessity for robust family and environmental support to aid in the return to daily activities. Regarding the clinical treatment administered to this patient, it is noteworthy that while initial measures such as steroid administration upon admission and rituximab as an adjunct to reduce the risk of relapse were employed, studies have also indicated symptomatic management with benzodiazepines, as well as surgical intervention in cases with probable immune-mediated paraneoplastic etiology [9,10].

## Conclusions

In conclusion, this case sheds light on two important knowledge gaps in the understanding and management of AE, particularly in patients positive for anti-NMDAR antibodies. Firstly, the rarity of catatonia as an initial presentation in young patients underscores the need for further research and awareness regarding the varied clinical manifestations of the disease beyond the pediatric population. Secondly, the necessity for a comprehensive, multidisciplinary treatment approach is emphasized, encompassing not only medical interventions but also psycho-cognitive therapy and strong familial support to facilitate patients' reintegration into daily life. This case underlines the importance of ongoing research and collaboration in refining treatment strategies for autoimmune encephalitis patients to improve outcomes and quality of life.
